# Infants of Diabetic Mothers and Associated Complications in the Neonatal Intensive Care Unit

**DOI:** 10.7759/cureus.76137

**Published:** 2024-12-21

**Authors:** Hussain A Al Ghadeer, Ahad A Mohamed, Mariam A Alali, Kulthum A Al Mahdi, Sajeda M Almishal, Tawfiq M Aljubran, Abdulelah A Alneamah, Roaa S Alduhmush, Mohammed J Alobaid, Tarfh S Alsaad, Hayam S Almoagal, Abdullah M Albuali, Mudhawi F Alsuliman, Noor A Althafar, Essa F Al-Shaalan

**Affiliations:** 1 Pediatrics, Maternity and Children Hospital, Al-Hofuf, SAU; 2 Pediatrics, Johns Hopkins Aramco Healthcare, Dhahran, SAU; 3 Pediatrics, Dr. Sulaiman Al Habib Medical Group, Khobar, SAU; 4 Pediatrics, King Faisal University, Al-Hofuf, SAU; 5 Medicine, King Faisal University, Al-Hofuf, SAU

**Keywords:** alahsa, diabetes, gestational diabetes, maternal, mothers, neonatal, outcome, pregnancy, saudi arabia

## Abstract

Background

The incidence of pregnancy-associated diabetes has increased in recent decades, leading to neonatal adverse outcomes like metabolic and hematologic disorders, respiratory distress, cardiac disorders, and neurologic impairment. Macrosomia, a common consequence of diabetes, is influenced by maternal blood glucose levels, impacting adverse neonatal outcomes.

Aim

The current study aimed to assess the neonatal and maternal outcomes of the infants of diabetic mothers.

Methods

An observational retrospective study was conducted among infants of diabetic mothers at Maternity and Children Hospital, Saudi Arabia, from 2022 to 2023. The data included socio-demographic details, diabetes-related information, and maternal and neonatal outcomes.

Results

A study of 400 mothers aged 18-40 years found that 54.3% had 1-4 previous pregnancies, while 35.5% had more than four. The majority had gestational diabetes mellitus (GDM), with 25.5% having diabetes for more than five years. The most common complications were preeclampsia (7.5%), polyhydramnios (6%), UTI (5.3%), PROM (4.3%), and pregnancy-induced hypertension (4.3%). The majority had no complications, while 12.5% of neonates had respiratory and metabolic complications.

Conclusion

The study found that most women with gestational diabetes had previously been diagnosed with diabetes mellitus (DM) and are multiparous, with Caesarean delivery being the dominant mode. While maternal complications were seen in only less than one-third of mothers, neonatal complications were noted in 12.5%.

## Introduction

Globally, gestational diabetes mellitus (GDM) affects 2% to 9% of pregnant women and is characterized as "any level of glucose intolerance first recognized during pregnancy." The placenta during pregnancy releases certain diabetogenic hormones such as growth hormone, corticotropin-releasing hormone, human placental lactogen, prolactin, and progesterone [1,2]. Additionally, pregnancy is linked to insulin resistance. When this insulin resistance combines with inadequate pancreatic function, the risk of developing GDM rises [3,4].

Over the past 50 years, preconceptional care, GDM screening, and management have advanced in high-income countries [5]. However, access to high-quality prenatal care for the diagnosis and treatment of GDM is frequently limited in low- and middle-income nations. As a result, although this point is not well documented, the prenatal and neonatal burden of GDM may be paradoxically higher in these countries [6].

Diabetes during pregnancy is a major cause of pregnancy-related maternal morbidities [7]. Maternal-fetal and neonatal complications, such as macrosomia, neonatal hypoglycemia, hyperbilirubinemia, shoulder dystocia, birth trauma, and stillbirth, are common among women with GDM [8-11]. Recent studies have shown an increased risk of congenital anomalies, with multiple anomalies present in 13.6% of diabetes cases and 6.1% of non-diabetes cases [12-14]. Offspring born to mothers with diabetes in pregnancy have a 50% increased risk of congenital anomalies of the kidney and urinary tract (CAKUT). Preventing or eliminating gestational diabetes could eliminate 2.0% to 3.7% of CAKUT cases in the USA. Additionally, newborns of mothers with diabetes have a 47% higher prevalence of congenital abnormalities and cardiac and CNS anomalies [14].

Reference to a local systematic review study, the highest GDM prevalence was observed in Qatar (20.7%, 95% CI, 15.2-26.7%; 19 studies), followed by 15.5% in Saudi Arabia (95% CI, 12.6-18.8%; 48 studies) and 13.4% in the United Arab Emirates (95% CI, 9.4-18.0%; 14 studies) [15]. The current study aims to assess the impact of pre-pregnancy and gestational diabetes on mothers and their neonates.

## Materials and methods

A record-based observational retrospective study was carried out on infants born to mothers with diabetes who were admitted to the neonatal care unit at the Maternity and Children Hospital in Al-Hofuf, Saudi Arabia, between 2022 and 2023. The study included mothers with both GDM and pregestational DM (type 1 and type 2). Maternal data included age, parity, diabetes type and duration, diabetes treatment, and delivery method. A pregnant woman between 20 and 24 weeks of pregnancy was considered to have GDM if, following an oral glucose tolerance test with a 50 g glucose bolus, she tested positive for two or more of the following cut-off points: fasting blood sugar of 100 mg/dl; one-hour sugar of 190 mg/dl; two-hour sugar of 165 mg/dl; and three-hour sugar of 145 mg/dl. Gestational age (GA) and birth weight (BWT) were among the infants' attributes listed in the second set of data. The complications that were evaluated included birth injury, neurological, hematological, metabolic, respiratory, cardiac, and congenital anomalies. Additional variables included random blood sugar readings according to hospital protocol, as well as serum total calcium, total and direct bilirubin, magnesium levels, and hematocrit to assess for polycythemia.

Data analysis

The data were collected, reviewed, and then transferred to IBM SPSS Statistics for Windows, Version 26 (Released 2019; IBM Corp., Armonk, New York, United States). All statistical methods used were two-tailed with significance defined as a p-value ≤ 0.05. Descriptive analysis for categorical data was done using frequencies and percentages, whereas numerical data were presented as mean with standard deviation. All graphs were created using Microsoft Excel software (Microsoft Corporation, Redmond, United States). Frequency tables for mothers' bio-demographic data, obstetric history, and pregnancy outcome were created. Also, neonatal and maternal complications were tabulated and graphed. Cross-tabulation for showing factors associated with maternal and neonatal complications among diabetic mothers using the Pearson chi-square test and exact probability test for small frequency distributions.

## Results

Table 1 presents data on 400 eligible participants included in the study. The mothers' ages ranged from 18 to over 40 years, with a mean age of 34.2 ± 11.9 years old. Exactly, 217 (54.3%) mothers had 1-4 previous pregnancies while 142 (35.5%) had more than four, and 41 (10.3%) were primigravida. Also, 276 (69%) gave previous 1-4 births, 83 (20.8%) gave more than four, and 41 (10.3%) were primipara. A total of 148 (37%) mothers received preconception care. About 123 (30.8%) mothers reported a history of neonatal death, with the most reported causes being uncontrolled DM (11.4%), congenital heart disease (CHD) (8.1%), congenital anomalies (8.1%), and prematurity (3.3%), while the cause was unknown for most of the mothers (60.2%; 70).

**Table 1:** Bio-demographic and obstetric data of study mothers in Saudi Arabia (n=400) DM: diabetes mellitus

Bio-demographic data	Number	Percentage
Maternal age in years		
18-30	130	32.5
30-40	196	49.0
>40	74	18.5
Gravidity		
Primigravida	41	10.3
1-4	217	54.3
>4	142	35.5
Parity		
Primipara	41	10.3
1-4	276	69.0
>4	83	20.8
Preconception care		
Yes	148	37.0
No	252	63.0
History of neonatal death		
Yes	123	30.8
No	277	69.3
If yes, what is the cause?		
Unknown	74	60.2
Uncontrolled DM	14	11.4
Congenital heart disease	10	8.1
Congenital anomalies	10	8.1
Prematurity	4	3.3
Molar pregnancy	2	1.6
Neurological anomalies	2	1.6
Preeclampsia	2	1.6
Vascular abnormality	2	1.6
Ectopic pregnancy	1	.8
Ruptured membrane	1	.8
Syndromic	1	.8

Table 2 shows diabetes and medical data among study mothers in Saudi Arabia. A total of 244 (61%) mothers had GDM, 102 (26%) had type II DM, and 54 (13.5%) had type I DM. As for the duration of diabetes, 94 (25.5%) were diabetic for more than 5 years, 43 (10.8%) were diabetic for 1-5 years, and 20 (5%) were diabetic for less than one year, while 143 (35.8%) had their first GDM, and 100 (25%) had previous GDM. As for the regimen of treating DM, 156 (39%) were on diet only, 144 (36%) had insulin only, and 66 (16.6%) were on medications. Diabetes was controlled among 222 (55.5%) mothers. As for other comorbidities, the most reported included hypertension (5%), hypothyroidism (4%), epilepsy (2.8%), and sickle cell disease (SCD) (2%). The vast majority of the mothers (73.8%; 295) had no comorbidities.

**Table 2:** Diabetes and medical data among study mothers in Saudi Arabia (n=400) GDM: gestational diabetes mellitus; DM: diabetes mellitus

Medical history of the mothers	Number	Percentage
Type of DM		
DM type 1	54	13.5
DM type 2	102	25.5
Gestational DM	244	61.0
Duration of DM		
First GDM	143	35.8
Previous GDM	100	25.0
<1 year	20	5.0
1-5 years	43	10.8
>5 years	94	23.5
Treatment of diabetes		
Diet only	156	39.0
Insulin only	144	36.0
Medication and insulin	34	8.5
Medications only	66	16.6
Control of DM		
Controlled	222	55.5
Uncontrolled	178	44.5
Chronic diseases other than DM		
None	295	73.8
Hypertension	20	5.0
Hypothyroidism	16	4.0
Epilepsy	11	2.8
Sickle cell disease	8	2.0
Depression	7	1.8
Hyperthyroidism	7	1.8
G6PD deficiency	6	1.5
Inflammatory bowel disease (IBD)	6	1.5
Systemic lupus erythematosus (SLE)	6	1.5
Asthma	6	1.5
Hepatitis	3	0.8
Ischemic heart disease	3	0.8
Bipolar disease	1	0.3
Breast cancer	1	0.3
Hyperlipidemia	1	0.3
Iron deficiency anemia	1	0.3
Rhumatoarthritis	1	0.3
Thalassemia	1	0.3

Table 3 shows pregnancy outcomes among infants of diabetic mothers in Saudi Arabia. Half of the newborns were males (50%), and 188 (47%) were delivered normally (NVD). As for GA, 284 (71%) children were full-term. Considering BWT, it was normal among 290 (72.5%), low among 55 (13.8%), and high among 55 (13.8%) babies.

**Table 3:** Pregnancy outcomes among infants of diabetic mothers in Saudi Arabia (n=400) VLBW: very low birth weight; LBW: low birth weight; NBW: normal birth weight; HBW: high birth weight

Pregnancy outcome	Number	Percentage
Child gender		
Male	200	50.0
Female	200	50.0
Mode of delivery		
Vaginal delivery	188	47.0
Cesarean section	212	53.0
Gestational age		
Very preterm (32 to 28 weeks)	1	0.3
Moderate preterm (32 to <34 weeks)	13	3.3
Late preterm (34 to <37 weeks)	102	25.5
Full term (37 to 42 weeks)	284	71.0
Birth weight		
VLBW (<1500 to 1000 g)	3	0.8
LBW (<2500 to 1500 g)	52	13.0
NBW (2500 to 3999 g)	290	72.5
HBW (≥4000 g)	55	13.8

**Figure 1:**
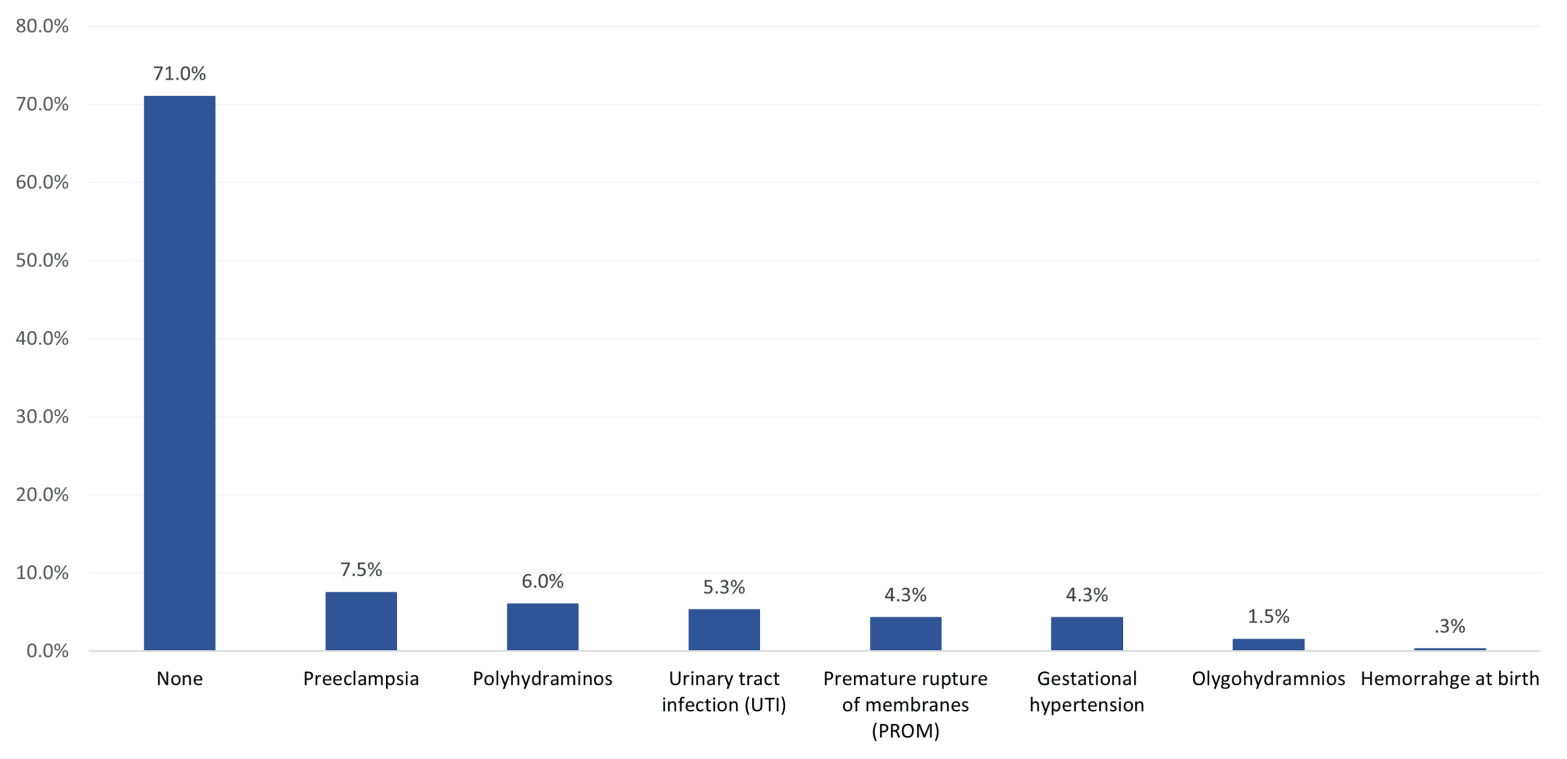
Maternal complications among mothers with diabetes in Saudi Arabia (n=400)

Regarding maternal complications (Figure 1), the most reported complications were preeclampsia (7.5%), polyhydramnios (6%), urinary tract infections (UTIs) (5.3%), premature rupture of membranes (PROM) (4.3%), and pregnancy-induced hypertension (4.3%). The vast majority of the study mothers (71%) had no complications.

Table 4 shows neonatal outcomes among infants of diabetic mothers in Saudi Arabia. Exactly 12.5% of the neonates of diabetic mothers had respiratory complications, mainly transient tachypnea (8.3%) and meconium aspiration syndrome (2.5%). Also, 21.5% of the neonates had metabolic complications, mainly hyperbilirubinemia (10.3%), hypoglycemia (7.5%), and hypocalcemia (2.3%). Only 3.2% of the neonates had neurological complications, mainly neonatal hypoxic-ischemic encephalopathy (1.8%) and spina bifida (1.3%). Likewise, 7.5% had hematological complications, mainly polycythemia (3.5%) and thrombocytopenia (4%). Congenital anomalies were detected among 5.2% of the neonates of diabetic mothers, including hydronephrosis (1.5%) and dysmorphic (TRISOMY 21) (1.6%). Birth injuries were detected among 5% of the neonates, including caput succedaneum (1.8%) and cephalohematoma (1.8%). Considering the cardiac outcome, 156 (39%) neonates had isolated CHD, and 113 (28.3%) had multiple CHD.

**Table 4:** Neonatal outcomes among infants of diabetic mothers in Saudi Arabia (n=400) CHD: coronary heart disease

Neonatal outcome		Number	Percentage
Respiratory	None	350	87.5
	Transient tachypnea of the newborn (TTN)	33	8.3
	Meconium aspiration syndrome (MAS)	10	2.5
	Respiratory distress syndrome (RDS)	6	1.5
	Hypoglycemia	1	0.3
Metabolic	None	314	78.5
	Hyperbilirubinemia	41	10.3
	Hypoglycemia	30	7.5
	Hypocalcemia	9	2.3
	Hypomagnesemia	8	2.0
	Intrauterine growth restriction (IUGR)	8	2.0
	Hypomagnesemia	7	1.8
	Macrosomia	1	0.3
Neurological	None	387	96.8
	Neonatal hypoxic-ischemic encephalopathy (HIE)	7	1.8
	Spina bifida	5	1.3
	Intraventricular hemorrhage (IVH)	1	0.3
Hematological	None	370	92.5
	Polycythemia	14	3.5
	Thrombocytopenia	16	4.0
Congenital anomalies	None	379	94.8
	Cleft palate/lip	1	0.3
	Dysmorphic (Goldenh syndrome)	1	0.3
	Hydronephrosis	6	1.5
	Dysmorphic (TRISOMY 21)	3	0.8
	Dysmorphic (TRISOMY 21) with duodenal atresia	3	0.8
	Single umbilical artery	2	0.5
	Gastroschisis	1	0.3
	Hypospadias	1	0.3
	Sacral agenesis, brain atrophy	1	0.3
	Solitary kidney	1	0.3
	Small left colon syndrome	1	0.3
Birth injury	None	380	95.0
	Caput succedaneum	7	1.8
	Cephalohematoma	7	1.8
	Caput succedaneum with subconjunctival hemorrhage	5	1.3
	Clavicular fractures	1	0.3
Cardiac outcome	Normal	131	32.8
	Isolated CHD	156	39.0
	Multiple CHD	113	28.3

**Figure 2:**
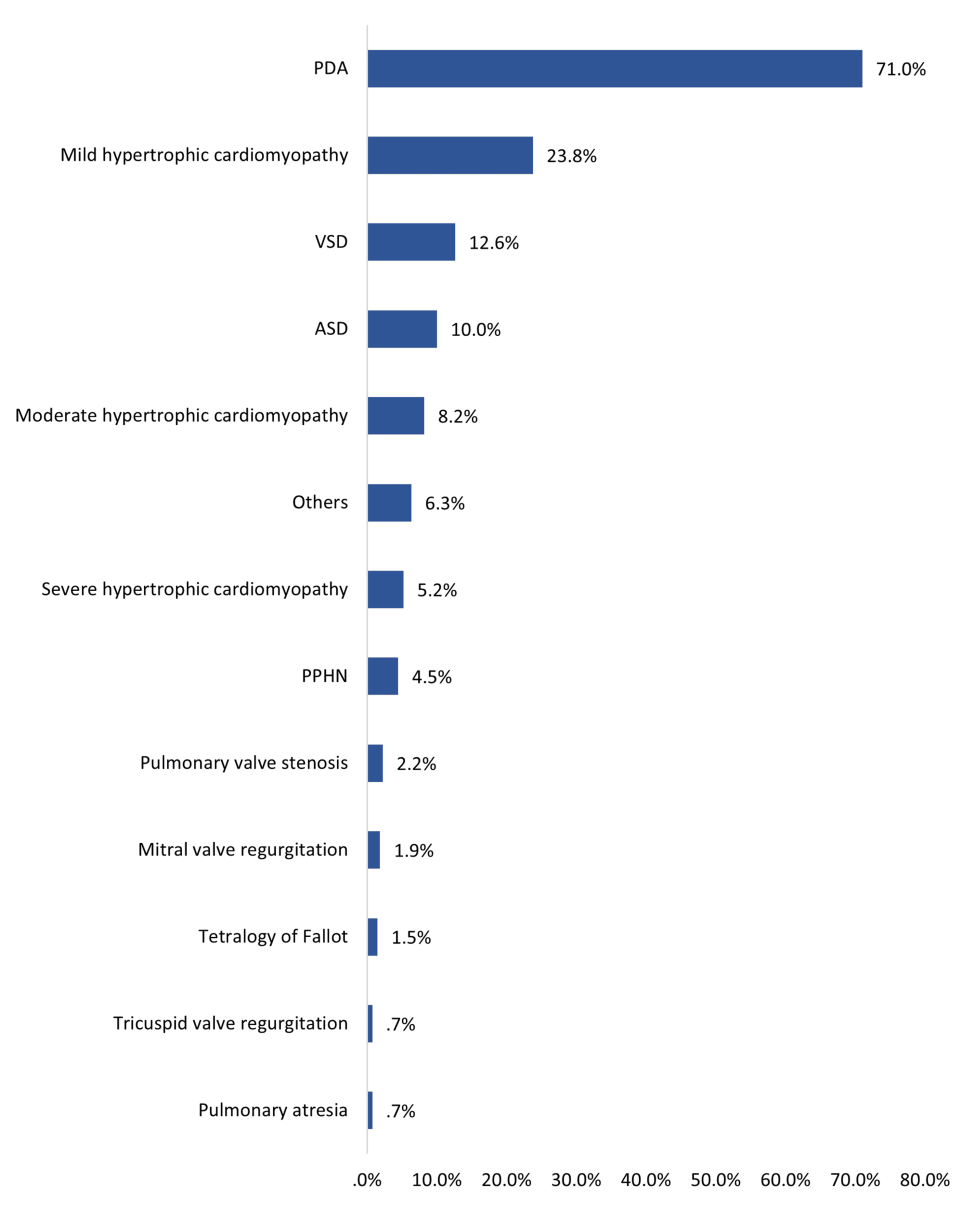
Types of congenital heart diseases among infants of diabetic mothers in Saudi Arabia (n=269) PDA: patent ductus arteriosus; VSD: ventricular septal defect; ASD: trial septal defect; PPHN: persistent pulmonary hypertension

Figure 2 shows types of CHDs among infants of diabetic mothers; the most reported included patent ductus arteriosus (PDA) (71%), mild hypertrophic cardiomyopathy (23.8%), and ventricular septal defect (VSD) (12.6%). The least reported cardiac anomalies were tetralogy of Fallot (1.5%), tricuspid valve regurgitation (0.7%), and pulmonary atresia (0.7%).

Table 5 shows factors associated with maternal complications among diabetic mothers in Saudi Arabia. A total of 37.4% of mothers with a history of neonatal death experienced complications, compared to 25.3% of those without such a history (p = 0.014). Also, 20.3% of mothers with controlled DM had complications compared to 39.9% of others with uncontrolled DM (p = 0.001). Other factors showed insignificant association with maternal complications history.

**Table 5:** Factors associated with maternal complications among infants of diabetic mothers in Saudi Arabia P: Pearson X^2^ test; ^: exact probability test; *p < 0.05 (significant) GDM: gestational diabetes mellitus; DM: diabetes mellitus

Factors	Maternal complications				p-value
	Yes (116; 29%)		No (284; 71%)		
	Number	Percentage	Number	Percentage	
Maternal age in years					0.227
18-30	45	34.6	85	65.4	
30-40	52	26.5	144	73.5	
>40	19	25.7	55	74.3	
Chronic diseases other than DM					0.910
Yes	30	28.6	75	71.4	
No	86	29.2	209	70.8	
History of neonatal death					0.014*
Yes	46	37.4	77	62.6	
No	70	25.3	207	74.7	
Type of DM					0.461
DM type 1	19	35.2	35	64.8	
DM type 2	31	30.4	71	69.6	
Gestational DM	66	27.0	178	73.0	
Duration of DM					0.663^
First GDM	37	25.9	106	74.1	
Previous GDM	29	29.0	71	71.0	
<1 year	5	25.0	15	75.0	
1-5 years	16	37.2	27	62.8	
>5 years	29	30.9	65	69.1	
Control of DM					0.001*
Controlled	45	20.3	177	79.7	
Uncontrolled	71	39.9	107	60.1	
Preconception care					0.482
Yes	46	31.1	102	68.9	
No	70	27.8	182	72.2	
Gravidity					0.422
Primigravida	9	22.0	32	78.0	
1-4	68	31.3	149	68.7	
>4	39	27.5	103	72.5	
Parity					0.564
Primipara	9	22.0	32	78.0	
1-4	83	30.1	193	69.9	
>4	24	28.9	59	71.1	

Table 6 shows factors associated with neonatal complications among diabetic mothers in Saudi Arabia. Neonatal complications were reported among 86% of mothers with diabetes for 1-5 years versus 65% of mothers with DM for less than one year and 72.7% of mothers with their first GDM (p = 0.047). Also, 83.6% of HBW infants experienced complications compared to 66.7% of others with VLBW (p = 0.048).

**Table 6:** Factors associated with neonatal complications among infant diabetic mothers, Saudi Arabia P: Pearson X^2^ test; ^: exact probability test; *p < 0.05 (significant) VLBW: very low birth weight; LBW: low birth weight; NBW: normal birth weight; HBW: high birth weight; GDM: gestational diabetes mellitus; DM: diabetes mellitus; PT: preterm

Factors		Neonatal complications				p-value
		Yes (314; 78.5%)		No (86; 21.5%)		
		Number	Percentage	Number	Percentage	
Maternal age in years	18-30	106	81.5	24	18.5	0.559
	30-40	150	76.5	46	23.5	
	>40	58	78.4	16	21.6	
Chronic diseases other than DM	Yes	77	73.3	28	26.7	0.133
	No	237	80.3	58	19.7	
History of neonatal death	Yes	101	82.1	22	17.9	0.241
	No	213	76.9	64	23.1	
Type of DM	DM type 1	44	81.5	10	18.5	0.255
	DM type 2	85	83.3	17	16.7	
	Gestational DM	185	75.8	59	24.2	
Duration of DM	First GDM	104	72.7	39	27.3	0.047*
	Previous GDM	80	80.0	20	20.0	
	<1 year	13	65.0	7	35.0	
	1-5 years	37	86.0	6	14.0	
	>5 years	80	85.1	14	14.9	
Control of DM	Controlled	176	79.3	46	20.7	0.672
	Uncontrolled	138	77.5	40	22.5	
Preconception Care	Yes	121	81.8	27	18.2	0.224
	No	193	76.6	59	23.4	
Gravidity	Primigravida	34	82.9	7	17.1	0.352
	1-4	174	80.2	43	19.8	
	>4	106	74.6	36	25.4	
Parity	Primipara	34	82.9	7	17.1	0.765
	1-4	215	77.9	61	22.1	
	>4	65	78.3	18	21.7	
Maternal complications	Yes	88	75.9	28	24.1	0.412
	No	226	79.6	58	20.4	
Gender	Male	154	77.0	46	23.0	0.465
	Female	160	80.0	40	20.0	
Gestational age	Very PT (32 to 28 weeks)	1	100.0	0	0.0	0.439^
	Moderate PT (32 to <34 weeks)	8	61.5	5	38.5	
	Late PT (34 to <37 weeks)	82	80.4	20	19.6	
	Full term (37 to 42 weeks)	223	78.5	61	21.5	
Birth weight	VLBW (<1500 to 1000 g)	2	66.7	1	33.3	0.048*^
	LBW (<2500 to 1500 g)	35	67.3	17	32.7	
	NBW (2500 to 3999 g)	231	79.7	59	20.3	
	HBW (≥4000 g)	46	83.6	9	16.4	

## Discussion

The goal of the current study was to assess maternal and neonatal outcomes for mothers with diabetes and related risk factors. Compared to infants born to women without DM, babies born to women with DM are more likely to have large gestational age (LGA) and to be born prematurely (PTB) [16-19]. Perinatal asphyxia, respiratory distress syndrome (RDS), hypoglycemia, hypocalcemia, polycythemia, transient hypertrophic cardiomyopathy [20,21], cardiovascular (CVS) and central nervous system (CNS) defects, hyperbilirubinemia, low iron stores, and macrosomia are among the neonatal complications that these infants are more likely to experience.

According to the current study, the majority of diabetic mothers were at least 30 years old, multipara, and multigravida. More than one-third of the diabetic mothers received preconceptional care because fewer than one-third had a history of neonatal death. Prematurity and uncontrolled DM with congenital defects were the most frequently reported causes of neonatal mortality. One-third of the study mothers had GDM for the first time, and one-fourth had recurrent GDM. GDM was the most common type of diabetes among the mothers.

Although fewer mothers were taking medication, over one-third of the mothers with diabetes were on dietary control, and roughly one-third were on insulin. Other comorbidities were absent in the majority of the mothers. Similar results were previously reported by Engelgau et al. [22], who found that the majority of pregnant women with diabetes had GDM, with an average age of 30. Additionally, Harris et al. [23] discovered that mothers with diabetes who had primarily recurrent GDM were older than 35. GDM was found in 39% of pregnant Saudi women, according to Alfadhli et al. [24]. Women with GDM were treated with diet, exercise, and insulin as necessary. The results of our study are in line with another study by Subki et al. [25], which found that pregnant women with diabetes had an average age of 31.3±6.7 years, an average gravidity of 4.0, and an average parity of 3.0.

Less than one-third of diabetic mothers experienced complications, primarily preeclampsia, polyhydramnios, UTI, PROM, and pregnancy-induced hypertension, according to the current study. The majority of studies evaluated the long-term complications of GDM in mothers, primarily the development of type II DM [26-30]. The results of the current study are consistent with another study by Preda A et al. [31], which found that the most common maternal complications were cesarean delivery, gestational hypertension, and preeclampsia, which were reported by over half of the mothers in the current study. Other studies evaluated maternal complications in women with a diagnosis of GDM, primarily preeclampsia and cesarean delivery [32-34].

According to the current study, the majority of babies were born at full term and with normal BWT. In contrast to the trend literature conclusion, less than one-fifth of the babies born to diabetic mothers had large BWTs [35-37]. A Japanese multicenter study that examined data from 2003 to 2009 found that the proportion of macrosomia in type 1 diabetes was 4.6%, while in type 2 diabetes, it was 5.0% [38]. This study is comparable to ours. Additionally, Mitanchez et al. [39] discovered that 5.9% of people with type 2 diabetes had macrosomia. The high rate of cesarean sections in diabetic conditions may have been influenced by the high proportion of macrosomia. because growth-related health problems are also a risk for neonates with macrosomia [40].

According to the current study, the majority of neonates born to mothers with diabetes had complications, primarily respiratory, metabolic, and cardiac issues (both isolated and multiple). Additionally, there were very few reports of congenital abnormalities among the neonates in the current study. Neonates with birth injuries and neurological complications were rare. Hypertrophic cardiomyopathy and PDA were the most frequently reported cardiac complications. Although the risk is lower than in women with pregestational diabetes, Balsells et al. [41] provide evidence that GDM is linked to a marginally elevated risk of congenital defects. Between 25% and 75% of babies born to mothers with diabetes have been found to have myocardial hypertrophy, a finding that has been documented in both pregestational diabetes and GDM [42,43]. According to Corrigan et al. [44], VSDs, truncus arteriosus, hypoplastic left heart syndrome, transposition of the great arteries, and double outlet right ventricle are the most frequently reported cardiac malformations. Additionally, there were reports of metabolic disorders such as hyperbilirubinemia [45,46], hypoglycemia [47,48], and hypocalcemia [49,50].

## Conclusions

The current study found that the majority of pregnant women with diabetes had GDM, predominantly due to recurrent episodes. Additionally, they were primarily multiparous and older than 30. The majority of neonates with cesarean deliveries had normal BWTs and GAs. Maternal complications were rare and linked to uncontrolled DM and a history of neonatal death. Neonatal complications, on the other hand, were common, and CVS and metabolic complications were the most common. High BWT and prolonged DM/recurrent GDM were substantially linked to the incidence of neonatal complications. For high-risk mothers with diabetes, appropriate screening and prenatal care are essential to lowering the burden of complications for both the mother and the unborn child. Pregnancy-related factors to be taken into account include prenatal blood glucose levels and obstetric history of neonatal death.
